# New Mechanism by Which Human Cytomegalovirus MicroRNAs Negate the Proinflammatory Response to Infection

**DOI:** 10.1128/mBio.00505-17

**Published:** 2017-04-18

**Authors:** Andrew D. Yurochko

**Affiliations:** a Department of Microbiology and Immunology, Center for Molecular and Tumor Virology, Louisiana State University Health Sciences Center, Shreveport, Louisiana, USA; b Center of Excellence in Arthritis and Rheumatology, Louisiana State University Health Sciences Center, Shreveport, Louisiana, USA; c Center for Cardiovascular Diseases and Sciences, Louisiana State University Health Sciences Center, Shreveport, Louisiana, USA; d Feist-Weiller Cancer Center, Louisiana State University Health Sciences Center, Shreveport, Louisiana, USA

**Keywords:** HCMV, IKK, NF-κB, cytokines, human cytomegalovirus, miRNA, proinflammatory, signaling

## Abstract

Viruses have evolved many novel mechanisms to promote infection and to mitigate the host cell response to that infection. In the article by M. H. Hancock et al. (mBio 8:e00109-17, 2017, https://doi.org/10.1128/mBio.00109-17), the authors describe a new mechanism by which human cytomegalovirus (HCMV) microRNAs (miRNAs; miR-US5-1 and miR-UL112-3p) negate the proinflammatory response to infection. The authors document that these two viral miRNAs downregulate the NF-κB response through direct targeting of the IKKα and IKKβ mRNAs, which in turn, through diminished IκB kinases (IKKs), block production of proinflammatory cytokines (interleukin-6 [IL-6], CCL5, and tumor necrosis factor alpha [TNF-α]). Because most signaling pathways that promote NF-κB activation and nuclear translocation ultimately converge on the activation of the IKK complex, this new study documents that HCMV can strongly dictate how infected cells respond to internal and/or external stimuli and thus positively influence the outcome of both lytic and latent infection.

## COMMENTARY

The NF-κB family is an important transcription factor family that was originally discovered as a regulator of the immunoglobulin kappa light chain promoter in B cells (1). NF-κB, as a common term for the NF-κB family, is a multimeric family of transcription factors that regulates numerous gene families and thus influences nearly all aspects of human health and, when dysregulated, human diseases. Structurally, the NF-κB family members contain a Rel homology domain sequence and functionally form hetero- or homodimeric complexes between the different family members (1). Research on this classic transcription factor family has shown that it is composed of members with transcriptional activation domains, c-Rel, RelA (p65), and RelB, and members with DNA binding potential, NF-κB1 (p105/p50) and NF-κB2 (p100/p52).

**FIG 1:**
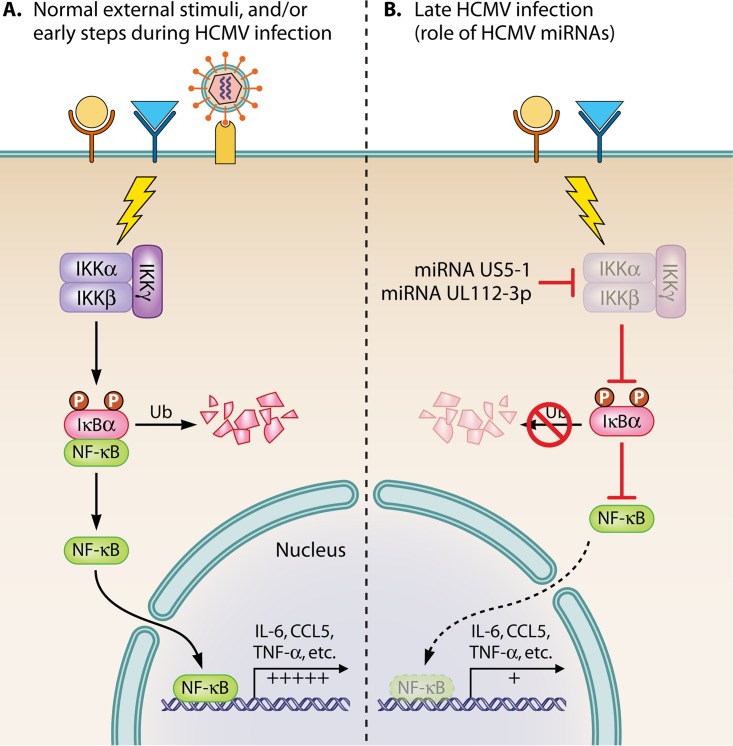
(A) Under normal conditions (for example, when IL-1β or TNF-α [represented by the circle and triangle] binds to its respective receptor, IL-1R or TNFR1 or TNFR2, or when HCMV initially binds to the target cell through glycoprotein binding to EGFR and cellular integrins), a signal transduction pathway is initiated converging on the IKK complex. This in turn results in serine phosphorylation of IκBα, leading to its ubiquitination and degradation, thus freeing NF-κB to translocate to the nucleus and transactivate high levels of proinflammatory cytokine genes such as IL-6, CCL5, and TNF-α. (B) Late during HCMV infection, once the US5-1 and UL112-3p miRNAs are produced, there will be the targeted reduction in the expression of IKKα and IKKβ gene products through binding of the miRNAs to the 3′ untranslated region of both mRNAs. For simplicity, in the figure this is represented as direct targeting of the IKKs. By decreasing IKKα and IKKβ expression, HCMV blocks or downregulates IκBα degradation. Consequently, only low levels of NF-κB can translocate to the nucleus, and thus, only low levels of proinflammatory cytokine genes will be transactivated. In addition to a reduction in the transactivation of proinflammatory genes due to the internal stimuli of the ongoing infection, there will also be diminished IKK signaling due to any external stimuli such as that provided by the addition of IL-1β or TNF-α to infected cells.

The NF-κB family members are controlled by various inhibitors termed IκBs (i.e., IκBα, IκBβ, etc.). In general, classic NF-κB, which is composed of the p50 and p65 subunits, is sequestered in the cytoplasm via its interaction with IκBα, which blocks the NF-κB nuclear localization signal (NLS). NF-κB is thus stored, preformed, in an inactive state. Following appropriate cellular activation, via cytokines, chemokines, growth factors, viral infection, etc., a downstream signaling pathway is initiated from the receptor-ligand engagement that usually culminates in the activation of the appropriate IκB kinase (IKK) complex (IKKα and IKKβ are the catalytic subunits, and IKKγ/NEMO is a structural regulator) and the phosphorylation of the critical serines, S32 and S36, on the IκBα molecules. These modifications allow ubiquitination and degradation of IκBα, thus freeing the NF-κB NLS and allowing it to translocate to the nucleus (Fig. 1A). Classically, this activation pathway is considered the canonical signaling pathway. Other pathways, such as the noncanonical pathway involving predominantly IKKα and the RelB/p52 heterodimer, are also known (1). Specificity and the nature of the distinct signaling pathways are determined by the unique receptor-ligand engagements and their corresponding downstream signaling components, the nature of the canonical versus noncanonical signaling pathway, and differences in activated family members, to name a few possibilities, along with differences in posttranscriptional modifications and the generation of additional transcriptional partners. Thus, the NF-κB signaling pathway is dynamic and complex with many regulatory control points.

The NF-κB family controls many aspects of human health, as it plays a key role during embryogenesis and controls multiple aspects of hematopoiesis and cellular development (1). Dysregulation of NF-κB is behind numerous pathologies, from cancer to cardiovascular diseases, to chronic inflammatory diseases, and to the diseases caused by multiple pathogens. In general, questions about NF-κB and its role in disease are centered on the dysregulation of NF-κB and how too much or too little is behind the various diseases.

In the paper by M. H. Hancock et al., the authors describe a novel mechanism by which human cytomegalovirus (HCMV) may alter the regulatory potential of NF-κB (2). As a backdrop, HCMV is a ubiquitous betaherpesvirus that is the leading cause of congenital infection (3, 4). Congenital infection results in significant neurological abnormalities that in turn can cause hearing loss, mental retardation, and motor deficits (3). HCMV is also a significant cause of morbidity and mortality in solid organ and bone marrow transplant recipients and in AIDS patients and is associated with various vascular diseases (3, 4). HCMV and NF-κB have long been known to be intimately linked (5). The HCMV major immediate early promoter, the central promoter that promotes the initiation of the viral life cycle and the promoter that is in use in many commercial/industrial plasmid constructs, etc., is molecularly and functionally defined as having NF-κB binding sites and as being positively regulated by NF-κB activation (3, 5). In addition, there is significant evidence that early in infection of multiple relevant cell types, NF-κB is activated and is essential for the beginning steps of the viral life cycle (3, 5, 6). The notice of a positive role for NF-κB activity is rooted in the regulatory role of NF-κB in transactivating key viral gene products, as well as in inducing the key cellular gene products required for infection, dissemination, and persistence in the many cell types infected by HCMV. Nevertheless, these data are counterintuitive because of the strong role that NF-κB plays in the induction of the innate and adaptive immune response to viruses (1). In a broad sense, the data suggest that the role and regulation of NF-κB are likely more complex than appreciated and that the virus takes advantage of these complexities and utilizes the positive aspects of NF-κB signaling to support lytic and latent infection while mitigating the negative aspects of the NF-κB-induced immune response. The study by Hancock et al. (2) attempts to address this conundrum.

As discussed above, HCMV induces NF-κB during the infection process. Functionally, NF-κB induction promotes necessary viral and cellular gene products. For the current project, the key regulated products are the NF-κB-dependent proinflammatory cytokines interleukin-6 (IL-6), CCL5, and tumor necrosis factor alpha (TNF-α). Early in infection, these and other proinflammatory cytokines could serve a positive or proviral role. They are generally considered to be induced by receptor-ligand engagement during the viral entry process (via gB and gH complex binding to appropriate cognate ligands—epidermal growth factor receptor [EGFR] and select integrins [6] [Fig. 1A]) and are thought to be key to viral dissemination in infected myeloid cells following primary and latent infection (7, 8). It has also been shown that in many cases there is a significant reduction in these types of proinflammatory cytokines late in infection, likely to diminish the deleterious effects of these cytokines on viral infection due to activation of the immune response. The mechanism for this late reduction in cytokine regulation has remained largely unresolved. However, in the study discussed the authors present novel data supporting a mechanism to account for the late inhibition of NF-κB-dependent cytokine expression.

The authors document that HCMV possesses two microRNAs (miRNAs), miR-US5-1 and miR-UL112-3p, that target the key IKK complex catalytic subunits IKKα and IKKβ. Targeting IKKα and IKKβ, the serine-threonine kinase members of the IKK complex, negatively regulates their expression and thus blocks the efficiency of the signaling convergence point for most of the pathways that activate NF-κB. By these actions, HCMV will be able to effectively limit production of proinflammatory cytokines due to either external or internal cellular stimuli (Fig. 1B). The authors showed directly in this study that by transfecting miR-UL112-3p and miR-US5-1 mimics, they could effectively downmodulate IKK protein levels. Molecularly, the 3′ untranslated regions of the IKK mRNAs were identified as the binding sites for the miRNAs. Through the use of various mutant viruses that were engineered to lack the miRNAs, the authors also showed an increase in the protein levels of the two IKKs, as well as a loss of control of late NF-κB signaling and loss of control of proinflammatory cytokine production, in cells infected with mutant HCMV. As stated/quoted by the authors, “these observations describe a mechanism through which HCMV miRNAs expressed late in the infectious cycle downregulate proinflammatory cytokine production to create a cellular proviral environment.”

This represents new data on the role that these miRNAs play during infection and mechanistically is an initial study showing how targeting the critical IKK complex can positively influence the outcome of HCMV infection. It is interesting to speculate that the miRNAs could also alter the ratio of the IKKs in addition to downmodulating IKKα and IKKβ levels in an infected cell. By altering the ratio of the IKKs, the virus could affect the canonical versus noncanonical pathways or could create differing combinations of possible dimeric IKKs that in either case could potentially favor the types of NF-κB family members produced during infection, which in turn would allow selective regulation of only those NF-κB-dependent promoters that favor viral infection and persistence.

In conclusion, the article by Hancock et al. (2) excitingly highlights functionally how two key HCMV miRNAs impact the signaling capabilities of infected cells, thus supporting lytic and/or latent infection. The data together are the first evidence suggesting how HCMV may control NF-κB-dependent proinflammatory events late in infection.
